# Complete genome sequencing and probiotic trait analysis of *Lacticaseibacillus rhamnosus* LR110, a human stool isolate from the NORDBIOTIC collection

**DOI:** 10.1128/mra.01116-24

**Published:** 2025-02-24

**Authors:** A. Szczepankowska, B. Cukrowska, T. Aleksandrzak-Piekarczyk

**Affiliations:** 1 Institute of Biochemistry and Biophysics, Polish Academy of Sciences 90863 , Warsaw, Poland; 2 Department of Pathomorphology, The Children Memorial Health Institute, Warsaw, Poland; University of Maryland School of Medicine, Baltimore, Maryland, USA

**Keywords:** probiotics, *Lactocaseibacillus rhamnosus*, lactobacilli, vitamin B1, lactose metabolism, bacteriocins, gut homeostasis, ornithine decarboxylase, putrescine biosynthesis, anti-obesity

## Abstract

We present the complete genome of *Lacticaseibacillus rhamnosus* strain LR110, a human stool isolate from the NORDBIOTIC strain collection. The genome consists of a 2,867,184-bp chromosome with 46.8% GC content. Genomic analysis revealed genes related to thiamine salvage, lactose metabolism, and putrescine biosynthesis, providing insight into the strain’s potential probiotic properties.

## ANNOUNCEMENT

*Lacticaseibacillus rhamnosus* is a rod-shaped, gram-positive lactic acid bacterium renowned for its probiotic properties (1). *L. rhamnosus* LR110 from the NORDBIOTIC collection originates from the feces of a healthy individual and has been deposited in the DSMZ Collection (DSM33794). LR110, along with other NORDBIOTIC strains, has been shown to alleviate symptoms of viral respiratory infections (2).

For this study, LR110 was taken directly from the NORDBIOTIC collection and cultured overnight from a single colony in MRS liquid medium (Oxoid) at 37°C under aerobic conditions. Cell pellets were pre-treated with lysozyme and mutanolysin prior to DNA extraction (3). DNA was isolated using a cetyltrimethylammonium bromide/lysozyme extraction protocol (4). The obtained DNA sample was sequenced using hybrid technology, and the resulting data are shown in Table 1.

**TABLE 1:** *L. rhamnosus* LR110 sequencing data

Description	Sequence ID
	LR110
GenBank accession no.	CP147912
Size (bp)	2,867,184
%GC	46.8
Coding sequences	2,599
Illumina raw data	
Number of sequences	557,312
Sequence length (bp)	294,330,379
Illumina trimmed data	
Illumina reads	535,134
Illumina data (nt)	250,676,561
Illumina coverage	56
ONT raw data	
Number of sequences	44,324
Sequence length (bp)	300,375,248
ONT trimmed data	
Number of sequences	32,659
Sequence length (bp)	295,055,194
*N*_50_	13,393
Average length	9,034.4
ONT coverage	76

Short-read sequencing was performed using the Illumina MiSeq platform (2 × 300 bp) with the NEB Ultra II FS library kit (New England Biolabs). Quality control and filtering of raw sequencing data were done using FASTQC (version 0.12.0) (5) and fastp (version 0.23.2) (6), resulting in 535,134 reads and 250,676,561 nt of final sequencing data.

For long-read sequencing, the GridION platform was used with the SQK-LSK109 native barcoding expansion kit (EXP-NBD103) and R9.4.1 flow cell (Oxford Nanopore Technologies). Basecalling was done using Guppy (version 6.1.3), adaptor removal with Porechop (version 0.2.4) (https://github.com/rrwick/Porechop), and quality control using NanoFilt (version 2.8.0) and NanoPlot (version 1.41.6) (7). This yielded 32,659 reads, with 295,055,194 bp of data and an *N*_50_ value of 13,393 bp.

Assembly of the nanopore reads, circularization, and contig rotation were performed using the Trycycler pipeline (version 0.5.3) (8) with multiple assemblers: Flye (version 2.9) (9), Unicycler (version 0.4.8) (10), Raven (version 1.8.1) (11), and Miniasm (version 0.3-r179) (12). Consensus sequences were corrected using Racon (version 1.5.0) (13) and Medaka (version 1.7.2) (14). Illumina short reads were then aligned to the assembled long-read contigs using Polypolish (version 0.5.0) (15) and POLCA (version 4.0.5) (16). All software was used with default parameters.

The resulting circular genome assembly was 2,867,184 bp in length, with a GC content of 46.8% and 188.0× coverage. Genome annotation, performed using the NCBI Prokaryotic Genome Annotation Pipeline (version 6.6) (17), identified 2,676 genes, including 2,599 coding sequences and 77 RNA genes. Notably, LR110 contains genes involved in the thiamine (vitamin B1) salvage pathway, important for short-chain fatty acid synthesis and gut microbiota homeostasis, as well as class IIb and IId bacteriocins, which together may contribute to modulating microbial dysbiosis and managing conditions, like inflammatory diseases, obesity, and allergy (2, 18). Additionally, components of the lactose-specific phosphotransferase system (EIIA and EIIBC) and complete tagatose pathway were identified, indicating the strain’s potential for efficient lactic acid production from lactose. Moreover, a gene encoding an ornithine decarboxylase (EC:4.1.1.17) involved in putrescine biosynthesis was detected, which has been linked to beneficial effects in patients with obesity and type 2 diabetes (19).

## Data Availability

Complete sequencing data have been deposited in GenBank under BioProject PRJNA1071652 and BioSample SAMN39639086. The whole genome sequence is available under accession number CP147912. Illumina SRA reads are available under accession number SRX23536627, and Oxford Nanopore SRA reads under accession number SRX23536626.
